# ABO incompatible living donor liver transplant with antibody titer of 1:4: First case report from Pakistan

**DOI:** 10.1016/j.amsu.2022.104463

**Published:** 2022-08-19

**Authors:** Abdul Wahab Dogar, Kaleem Ullah, Hafiz Bilal, Muhammad Shahzad Sarwar, Shams Uddin, Sidhant Ochani, Syed Hasnain Abbas

**Affiliations:** a Organ Transplantation and HBP Department, Pir Abdul Qadir Shah Jeelani Institute of Medical Sciences, Gambat, Pakistan; b Bone Marrow Transplant Department, Pir Abdul Qadir Shah Jeelani Institute of Medical Sciences, Gambat, Pakistan; c Khairpur Medical College, Khairpur Mir's, Pakistan

**Keywords:** ABO-Incompatible, Liver transplantation, Living liver donor, Case report

## Abstract

**Introduction and importance:**

The most common reason for live liver donor rejection is ABO incompatibility. With breaching this incompatibility barrier, probably an additional 25%–35% of liver transplantation (LT) procedures would become possible. Also, ABOi-LT can be lifesaving in acute settings. Initially, ABOi-LT reported a poor prognosis secondary to antibody-mediated rejection (AMR) which is more common in ABOi allograft recipients. AMR may be avoided by desensitization. Various desensitization protocols are practiced globally, however, there is no consensus available on the optimal desensitization protocol for the ABOi-LT. The ABO-incompatible (ABOi) can expand the liver donor pool tremendously. We report the first case of ABO incompatible-liver transplantation (ABOi-LT) from Pakistan.

**Case presentation:**

A 48 years old male, presented with decompensated liver diseaseand hepatocellular carcinoma secondary to HCV infection. LT was advised as the optimal modality of treatment. Due to the non-availability of a compatible donor, ABOi-LT was planned.His daughter agreed to donate.Pre-LT desensitization was started on the 23rd-day pre-LT with intravenous (I/V) rituximab 700 mg/body (375 mg/m^2^) along with I/V Bortezomib 2mg (1.3 mg/m^2)^. Bortezomib was repeated subcutaneously (S/C) on the 20th, 16th, and 13th days pre-LT. One week before LT oral Mycophenolate mofetil 500 mg and Tacrolimus 1 mg were started twice daily. Therapeutic plasmapheresis was done on the 5th, 3rd, and 1st-day pre-LT. Per-operatively, Basiliximab was administeredI/V with a dose of 0.8 gm/kg during the anhepatic phase. Anti-A & Anti-B titer level was determined on the 5th day before plasmapheresis and repeated on the 2nd and 1st-day pre-LT. Then post-LT plasmapheresis was done onthe 15th day and at 3 months. The CD 19 activity was determined one day before LT and on the 15th-day post-LT. His LT was performed uneventfully and was discharged on the 15th postoperative day (POD). However, on the 26th POD, he was diagnosed with left subclavian vein thrombosis which was treated successfully with anticoagulation therapy for 6 months. Till the last follow up patient is doing well.

**Clinical discussion:**

Desensitization is the removal of preformed anti-ABO antibodies and depleting serum B cells production. Antibody-mediated rejection irreversibly damages the graft and predisposes it to graft failure. The prognosis of ABOi-LT has dramatically improved since the introduction of desensitization protocols.

**Conclusion:**

Antibody-mediated rejection may be avoided by desensitization. The intravascular infusion therapies and splenectomy can be omitted from the desensitization protocol. ABO-i LT can tremendously increase the liver donor pool.

## Highlights

• Antibody-mediated rejection could be avoided by desensitization.
• There is no consensus available on a standardized ABOi-LDLT desensitization protocol.
• We report the first case of ABOi-LDLT from Pakistan with an antibody titer of 1:4.
• We used bortezomib along with rituximab and plasmapheresis as a desensitization protocol.
• In our case, we managed to reduce the anti-body titer to as low as 1:4 before the transplant.

## 1 Introduction

Living donor liver transplantation (LDLT) is an established alternative to deceased donor transplantation [1,2]. The common reason for donor rejection during LDLT donor screening is an ABO mismatch [3]. However, by breaching this barrier, approximately an additional 25–35% of LDLT procedures may become possible. Also, ABO incompatibility liver transplantation (ABOi-LT) can be a lifesaving procedure in acute settings [4]. Starzl was the first one who reported ABOi-LT in 1969 [5].

Antibody-mediated rejection (AMR) has been rarely reported in ABO-compatible recipients. However, it is more common in ABOi allograft recipients due to higher anti-A and anti-B isoagglutinin titers. The ABOi-LT reported an initially poor prognosis secondary to the higher incidence of AMR. It irreversibly damages the hepatic parenchyma and intrahepatic bile ducts and predisposes to graft failure [5,6].

A high anti-ABO antibody titer count is considered a major threat to AMR [7]. AMR can be avoided by desensitization i.e. procedure of removal of preformed anti-ABO antibodies by plasma paresis and depleting serum B cell production by various immunological medications. Pre and post-transplant various combinations of therapies are practiced as a part of desensitization protocol i.e., anti-CD 20 monoclonal antibodies, plasma exchange, intravenous immunoglobulin, portal/hepatic arterial prostaglandins infusion, splenectomy, and high doses various immunosuppressant drugs. However, most of the recently simplified protocols include preoperative plasma pheresis and anti-CD 20 monoclonal antibodies [[6], [7], [8], [9], [10]]. Repeated Plasma pheresis before LT rapidly lower anti-ABO antibody titers till it reaches a level considered to be safe for LT [[11], [12], [13]]. The prognosis of ABOi-LT has drastically improved since the introduction of desensitization with plasma paresis and anti-CD 20 monoclonal antibody i.e., rituximab [11].

There is no consensus available on the optimal desensitization protocol for ABOi-LDLT. Also, the preoperative antibody titer cut-off level that precludes LT has not yet been fully determined. Furthermore, the preoperative antibody titer level linked to AMR remains debatable. We report our first experience of ABOi-LDLT with an antibody titer of 1:4. We used bortezomib along with rituximab and plasma paresis as a desensitization protocol. In our case, we managed to reduce the anti-B titer to as low as 1:4 before LT.This case has been reported in line with the SCARE 2020 Criteria [14].

## 2 Case Presentation

A 48 years old male, having no co-morbidities, with clinical decompensated ascites secondary to HCV-related chronic liver disease. His family history was not significant for any illness, and he didn't have any significant past social and drug history, or history of any kind of allergies. His BMI was 26.8 kg/m^2^, Eastern Cooperative Oncology Group (ECOG) performance status of 0, Child Score of B-8, and Model for End-Stage Liver Disease(MELD) of 17. His HCV quantitative PCR showed no viral load. His CT scan abdomen showed cirrhotic liver morphology, segment VIII lesion measuring 3.7 x 3.4 × 3.0 cm suggestinghepatoma without vascular invasion or extrahepatic involvement. His Alpha-fetoprotein level was 1.1. His blood group was O-positive. LDLT was offered as the optimal modality of treatment.

In Pakistan, LDLT is commonly practiced, and according to the local human organ transplant act (HOTA), only blood relatives are allowed to donate. Unfortunately, due to the non-availability of a suitable ABO-compatible family donor, ABOi-LDLT was offered. His daughter was physically fit and she agreed to donate. She satisfied our donor criteriabut was having an incompatible blood group (B positive).

The benefits and pitfalls of the incompatible LDLT procedure were conveyed to the patient and family. Details of the donor surgical procedure, possible morbidity, and mortality of both donor and recipients were explained. Complete insight into the magnitude of the surgery and self-volunteered were confirmed in privacy.

A multidisciplinary team meeting was called for the discussion of the management plan. The team included hepatobiliary surgeons, hematologists, immunologists, gastroenterologists, anesthesiologists, and critical care specialists. Donor workup was done with detailed history, clinical examinations, and laboratory investigations including thrombophilia workup. MRCP for biliary and triphasic CT scans was performed to delineate the vascular anatomy, volumetry, and graft characteristics.

Pre-operative desensitization of the recipient was started on the 23rd-day pre-LT with intravenous (I/V) rituximab 700 mg/body (375 mg/m^2^) along with I/V Bortezomib 2mg (1.3 mg/m^2)^. Bortezomib was repeated subcutaneously onthe 20th, 16th, and 13th days pre-LT with the same dose. One week before the transplant oral Mycophenolate mofetil 500 mg and Tacrolimus 1 mg were started twice daily. Therapeutic plasmapheresis was done on the 5th, 3rd, and 1st-day pre-LT. On the operative day, I/V Basiliximab 0.8 gm/kg was administered during the hepatic phase. Anti-A & Anti-B titer level was determined on the 5th day before the 1st session of plasmapheresis and repeated on the 2nd and 1st day pre-LT.Then post-LT plasmapheresis was done onthe 15th day and 3 months. The CD 19 activity was determined one day before LT and on the 15th-day post-LT. The detailed protocol and laboratory parameters are given below in Table 1, Table 2.

**Table 1:** Showing pre-operative desensitization protocol.

Protocol Day	Desensitization Therapy	Anti-A & Anti-B titer level and CD-19 count
Pre-LT:day-23	Inj. Rituximab 375 mg/m^2^ = 700mg I.V slow	
	Inj. Bortezomib 1.3 mg/m^2^ = 2mg I.V slow	
Pre-LT:day-20	Inj. Bortezomib 1.3 mg/m^2^ = 2mg Subcutaneous(S/C)	
Pre-LT:day −16	Inj. Bortezomib 1.3 mg/m^2^ = 2mg S/C	
Pre-LT:day −13	Inj. Bortezomib 1.3 mg/m^2^ = 2mg S/C	
Pre-LT:day-7	Tab. Mycophenolate mofetil 500 mg twice daily	
	Tab. Tacrolimus 1 mg twice daily	
Pre-LT:day-5	Therapeutic plasmapheresis	Anti-A & Anti-B titer level = 1:32
Pre-LT:day-3	Therapeutic plasmapheresis	Anti-A & Anti-B titer level = 1:16
Pre-LT: day-1	Therapeutic plasmapheresis	Anti-A & Anti-B titer level = 1:4
		CD 19 activity = 1.6%
Operative day (day-0)	LT procedure performed	
	Inj. Basiliximab 0.8 gm/kg I.V (During anhepatic phase)	
Post-LT:day-15		Anti-A & Anti-B titer level = 1:16
		CD 19 activity = < 3%
Post-LT:3 months		Anti-A & Anti-B titer level = 1:16

**Table 2:** Post-operative laboratory parameters of the patient.

Laboratory Investigations	Day 1	Day 3	Day 5	Day 7	Day 10	Day 12	Day 15	Reference values
WBC (Cells/mm^3^)	13.90	4.05	3.22	3.34	18.95	11.05	8.30	4000–11000
Hb gm/dl	10.2	7.2	8.3	10.2	11.2	10.6	10.1	11.5–15.0
Platelets (Cells/mm^3^)	61	24	21	27	85	112	130	150–400 × 10^9^
INR	1.71	2.21	1.79	2.04	1.58	1.78	1.28	
Albumin (g/dl)	2.0	2.9	2.6	2.2	2.1	2.0	2.2	3.4–5.0
Total bilirubin (mg/dl)	5.3	2.4	1.9	1.6	2.1	1.5	0.6	1.2–2.0
ALT (U/L)	87	70	74	104	82	67	44	<45
AST (U/L)	138	104	81	61	46	27	30	9.4–36
Creatinine mg/dl	0.70	0.50	0.50	0.50	0.50	0.80	0.60	0.5–1.3
Tacrolimus/mL	–	–	7.50	–	14.64	–	12.50	5–15

On day 0 LDLT was performed. The right lobe graft without MHV weighing 582 having a single right hepatic vein, right portal vein, single artery, and bile duct, GRWR of 0.70, was implanted through the piggy back technique. The surgery was uneventful. At the end of surgery cholangiogram for biliary anatomy and Doppler ultrasound for hepatic vasculature was normal.

Postoperatively the patient was shifted ventilated to ICU. Strict isolation was maintained throughout the postoperative course. He was extubated the next morning after confirmatory normal doppler ultrasound for hepatic vasculature. Peri-operative Antibiotic prophylaxis was done with injectable Tazobactum plus Piperacilline 4.5 g twice for five days. Tacrolimus was continued on the 1st postoperative day (POD) with a dose of 1mg twice, and the dose was gradually increased to maintain trough levels up to 8–12 ng/ml. Mycophenolate Mofetil 1 gm twice a day was also resumed on POD1. From POD 1–4 Methylprednisolone continued in tapering doses i.e., 100 mg on 1st POD, 80 mg on 2nd POD, 60 mg on 3rd POD, and 40 mg on 4th POD. While on 5th POD prednisone 20 mg per oral was started and continued for 2 months. Beyond 2 months the dose was tapered by 5 mg per week till the end of 3 months the steroid was tapered. Oral Trimethoprim 80 mg + Sulphamethoxazole 400 mg on an alternate day and oral fluconazole 200 mg once a day were also continued for 3 months.

The patient remained in ICU for 10 days while the total hospital stay was 15 days. He was discharged on the 15th postoperative day. He did not meet with any complications or adverse events during his hospital stay. The CMV PCR on the 7^th^day, 3, and 6 months were negative.

During this period, he was followed weekly for 4 weeks, 2 weekly for 2 months, monthly for 6 months, and 3 monthly from then. Post-operatively no infective complication was observed. However, on the 26th POD,he got left subclavian vein thrombosis which was treated successfully with anticoagulation therapy (Enoxaparin1mg/kg twice daily S/C for 6 months). Till the last follow up patient is doing well.

## 3 Discussion

Antibody-mediated rejection (AMR) is a refractory rejection that is more common in donor-specific antibody (DSA) positive cases or ABOi grafts. AMR irreversibly damages the graft and predisposes it to graft failure [6].

Initial experiments on animals by Professor Starzl labeled the liver “a privileged organ” as it demonstrated more resistance to acute rejection compared to the heart and kidney [5]. But Later on, Rego et al. [15] in 1987, reported hyperacute rejection in ABOi-LT. Also, Gugenheim et al. [16,17] reported a lower graft survival with ABOi-LT. In their series of 17 ABOi-LT, immunological damage was postulated as the primary cause of poor graft survival. They reported AMR in 6 out of 17 patients. They also recorded progressive cholangitis and a higher incidence of arterial thrombosis in ABOi grafts. Similarly, Sanchez et al. [18] also reported a higher incidence of biliary complications i.e., bile leak and cholangitis, hepatic artery thrombosis, and cellular rejection in ABOi-LT.

Pakistan is developing country, with heavy burden of liver disease. It's healthcare system is facing serious challenges. The underprivileged people in the country who needed LT faces various financial issues [19]. The desensitization procedure will futhur add cost to the overall LT procedure.

But, due to a scarcity of deceased donors in Asia, LDLT is prevalent here. In majorities of these countries, the live liver donor pool is limited only to family members due to legal constraints. Also, emotional non-related donation is prohibited here [3]. It is tough to have always an ABO-compatible family donor. To overcome this ABO barrier, we began swapping LDLT in ABO mismatch pairs i.e., A to B and B to A blood group. However, in the O blood group recipients swap was not possible. So, for such mismatched patients, we started ABOi- LT program. This was the first case in the country in which ABOI-LDLT was performed. ABO-i LT can tremendously increase the liver donor pool.

Our desensitization protocol for this particular case included rituximab, bortezomib, plasmapheresis, Basiliximab, tacrolimus, and mycophenolate mofetil. We neither performed splenectomy nor used local hepatic artery/portal vein infusion therapy in this particular case.

Rituximab, a monoclonal antibody, is effective against the CD-20 antigen on the B cells. Also, the CD-19 antigen, a surrogate marker of the extent of CD-20 depletion is expressed in B cells. Rituximab starts functioning within three days, and after three weeks enough B cells depletion occurs. We also administered rituximab three weeks before surgery and through CD-19 count estimation, we monitored B cell depletion. Along with rituximab, mycophenolate mofetil was started 7 days before surgery for plasma cells removal, as rituximab does not affect plasma cells [20]. However, removal of preformed antibodies was done with double-filtration plasmapheresis. First plasma separation from whole blood was performed, then substances were filtered out by processing with a plasma fractionator and the final filtrate was returned to circulation. Literature has not shown any effect of splenectomy on postoperative titers so was not performed [21]. We avoided the local vascular infusion therapies due to their doubtful role in preventing AMR in the presence of effective perioperative plasmapheresis and rituximab [22].

Regarding immunological complications the first four weeks are important, and if the patient escapes that, further complications are uncommon [23]. We did not experience perioperative infectious complications.

## 4 Conclusion

Antibody-mediated rejection may be avoided by desensitization. The intravascular infusion therapies and splenectomy can be omitted from the desensitization protocol. We encourage other LDLT centers in the country to start ABOi-LT, as it will tremendously increase the liver donor pool in the country.

## Ethical committee approval

Pir Abdul Qadir Shah Jeelani Institute of Medical Sciences. (Reference Number: IRB/22/67).

## Patients perspective

I was having abdominal distension and pain in the right side of my tummy for the last 6 months. I visited many doctors and ultimately,I landed in the liver transplant unit. After a thorough workup, my doctor offered me a liver transplant. Everything went well and I was taken care of. Now, I am feeling much better and I thank them all for their care.

## Financial disclosure

None.

## Author's contributions

AWD performed the procedure, critical revision, and overall supervision. All authors wrote the manuscript. KU did the study conception and design. HB and SS participated in data collection. SHA and SU did the literature review. Review editing was done by SO and KU. Referencing was done by HB and SS.

## Consent

Written informed consent was obtained from the patient for publication of this case report. A copy of the written consent is available for review by the Editor-in-Chief of this journal on request.

## Registration of research study

N/A.

## Guarantor

Kaleem Ullah.

## Provenance and peer review

Not commissioned, externally peer-reviewed.

## Declaration of competing interest

Authors have no conflict of interest.
